# Association between endometrial thickness before ovulation, live birth, and placenta previa rates in clomiphene citrate-treated cycles

**DOI:** 10.1016/j.xagr.2023.100161

**Published:** 2023-01-18

**Authors:** Shogo Nishii, Kenji Ezoe, Seiko Nishihara, Sachie Onogi, Kazumi Takeshima, Shinya Karakida, Junichiro Fukuda, Keiichi Kato

**Affiliations:** 1 Kato Ladies Clinic, Shinjuku, Tokyo, Japan; 2 Department of Obstetrics and Gynecology, School of Medicine, Showa University, Tokyo, Japan

**Keywords:** clomiphene citrate, endometrial thickness, live birth, proliferative phase, placenta previa, single embryo transfer

## Abstract

**BACKGROUND:**

Although a recent study reported that the pregnancy outcomes in the first trimester were more correlated with endometrial thickness on the day of the trigger than with endometrial thickness on the day of single fresh-cleaved embryo transfer, it remains unclear whether endometrial thickness on the day of the trigger can predict live birth rate after a single fresh-cleaved embryo transfer.

**OBJECTIVE:**

This study aimed to examine whether endometrial thickness on the trigger day is associated with live birth rates and whether modifying the single fresh-cleaved embryo transfer criteria to reflect endometrial thickness on the trigger day improved the live birth rate and reduced maternal complications in a clomiphene citrate–based minimal stimulation cycle.

**STUDY DESIGN:**

This was a retrospective study of the outcomes of 4440 treatment cycles of women who underwent single fresh-cleaved embryo transfer on day 2 of the retrieval cycle. From November 2018 to October 2019, single fresh-cleaved embryo transfer was performed when endometrial thickness on the day of single fresh-cleaved embryo transfer was ≥8 mm (criterion A). From November 2019 to August 2020, single fresh-cleaved embryo transfer was conducted when endometrial thickness on the day of the trigger was ≥7 mm (criterion B).

**RESULTS:**

A multivariate logistic regression analysis revealed that increased endometrial thickness on the trigger day was significantly associated with an improvement in the live birth rate after single fresh-cleaved embryo transfer (adjusted odds ratio, 1.098; 95% confidence interval, 1.021–1.179). The live birth rate was significantly higher in the criterion B group than in the criterion A group (22.9% and 19.1%, respectively; *P*=.0281). Although endometrial thickness on the day of single fresh-cleaved embryo transfer was sufficient, the live birth rate tended to be lower when endometrial thickness on the trigger day was <7.0 mm than when endometrial thickness on the day of the trigger was ≥7.0 mm. The risk for placenta previa was reduced in the criterion B group when compared with the criterion A group (4.3% and 0.6%, respectively; *P*=.0222).

**CONCLUSION:**

This study demonstrated an association of decreased endometrial thickness on the trigger day with low birth rate and a high incidence of placenta previa. A modification of the criteria for a single fresh-cleaved embryo transfer based on endometrial thickness may improve pregnancy and maternal outcomes.

**AJOG Global Reports at a Glance**

**Why was this study conducted?**

This study aimed to assess the correlation of endometrial thickness on the day of the trigger with the live birth rate and perinatal outcomes after a single fresh-cleaved embryo transfer in a clomiphene citrate (CC)–based minimal stimulation cycle.

**Key findings**

Increased endometrial thickness on the trigger day was significantly associated with improved live birth rates after a single fresh-cleaved embryo transfer. The risk of placenta previa was reduced in the criterion B group when compared with the criterion A group.

**What does this add to what is known?**

Modifying the criteria for a single fresh-cleaved embryo transfer based on endometrial thickness on the day of ovulation trigger in CC-based minimal stimulation cycles may improve the live birth rate and reduce the incidence of placenta previa.

## Introduction

The endometrium is a steroid hormone–responsive tissue that undergoes cyclical proliferation, differentiation, and exfoliation during the menstrual cycle.^1^^,^^2^ Endometrial epithelial cells proliferate in the presence of estrogen during the first half of the menstrual cycle (also known as the proliferative phase). Conversely, the endometrium ceases to proliferate under the combined action of estrogen and progesterone and initiates differentiation for the preparation of implantation during the secretory phase.^3^ Steroid-induced proliferation and differentiation switching is necessary for the transition to the receptive phase.^4^ These endometrial alterations can be predicted by measuring the endometrial thickness (EMT) in assisted reproductive technologies (ARTs). Several studies have indicated a correlation between pregnancy outcomes and EMT on the day of the maturation trigger and embryo transfer in the controlled ovarian stimulation (COS) cycle; specifically, a previous study has reported an association between a decrease in the EMT and a reduced likelihood of pregnancy.^5^, ^6^, ^7^, ^8^, ^9^, ^10^ Therefore, in most institutions, EMT on the day of oocyte maturation trigger and/or embryo transfer is often used as an effective predictor of pregnancy outcomes in the COS cycle.

Clomiphene citrate (CC) is a drug used in assisted reproduction, primarily for the minimal stimulation cycle in in vitro fertilization (IVF).^11^, ^12^, ^13^, ^14^, ^15^ It is a selective estrogen receptor modulator that promotes follicular development and final maturation in IVF. ART outcomes such as preimplantation development and pregnancy outcomes in the CC-based minimal stimulation cycle are similar to those reported in the COS cycle.^12^^,^^16^ In contrast, CC impacts endometrial proliferation before ovulation by inhibiting estradiol binding to the receptor and adversely affecting endometrial differentiation because of the decline in sensitivity to progesterone.^17^ Therefore, endometrial function may be impaired in the CC-based minimal stimulation cycle.^18^^,^^19^ Considering these side effects, EMT should be monitored more closely in the CC-based minimal stimulation cycle than in other stimulation cycles. In our clinic, we performed single fresh-cleaved embryo transfers (SFCT) in the CC-based minimal stimulation cycle when EMT on the day of SFCT was ≥8 mm. A recent study reported that the ongoing pregnancy rate was more significantly correlated with EMT on the day that ovulation is triggered than with EMT on the day of SFCT.^20^ However, it remains unclear whether EMT on the day of the trigger can predict the live birth rate after SFCT. This study aimed to assess the correlation of EMT on the day of the trigger with the live birth rate after SFCT in the CC-based minimal stimulation cycle. Furthermore, we examined whether the modification of the SFCT criteria based on the EMT on the day of trigger improved the live birth rate and maternal outcomes after SFCTs in the CC-based minimal stimulation cycle.

## Materials and Methods

### Ethics approval

All procedures followed were in accordance with the ethical standards of the responsible committee on human experimentation (institutional and national) and were in accordance with the Helsinki Declaration of 1964 and its later amendments. This study was a retrospective cohort study approved by the institutional review board of Kato Ladies Clinic (approval number 21-9). Written informed consent for the retrospective analysis of deidentified data was obtained from all patients undergoing IVF treatment at the center.

### Study patients

The outcomes of 4440 treatment cycles of women (n=4440) who underwent oocyte retrieval during the study period during a CC-based minimal stimulation cycle were retrospectively analyzed. In this study, each patient was included only once in the analysis. The participants were scheduled for SFCT on day 2 of the retrieval cycle at the Kato Ladies Clinic between November 2018 and August 2020. From November 2018 to October 2019, criterion A was used: SFCT was performed when EMT was ≥8 mm on the day of SFCT (Figure). When EMT was <8 mm on the day of SFCT, the cleaved embryos were vitrified and transferred in the subsequent cycles. From November 2019 to August 2020, we applied the new criterion B based on the previous report that the ongoing pregnancy rate was significantly lower when EMT was <7 mm on the day of the trigger than when EMT was ≥7 mm.^20^ SFCT was performed when EMT was ≥7 mm on the day of the trigger. When EMT was <7 mm on the day of the trigger, oocyte retrieval was conducted, and the obtained embryos were used for the frozen embryo transfers in the subsequent cycles. The pregnancy outcomes, including live birth rate, after SFCT based on the 2 different criteria were retrospectively analyzed. Maternal outcomes were obtained from a questionnaire filled out by the patients after the infant's 1-month examination. Pregnancy complications were diagnosed in maternity hospitals. All pregnant women were invited to respond to the questionnaire at 9 weeks of gestation, during the second trimester, and after delivery. If they did not respond, we conducted a follow-up regarding their outcomes.

**Figure:**
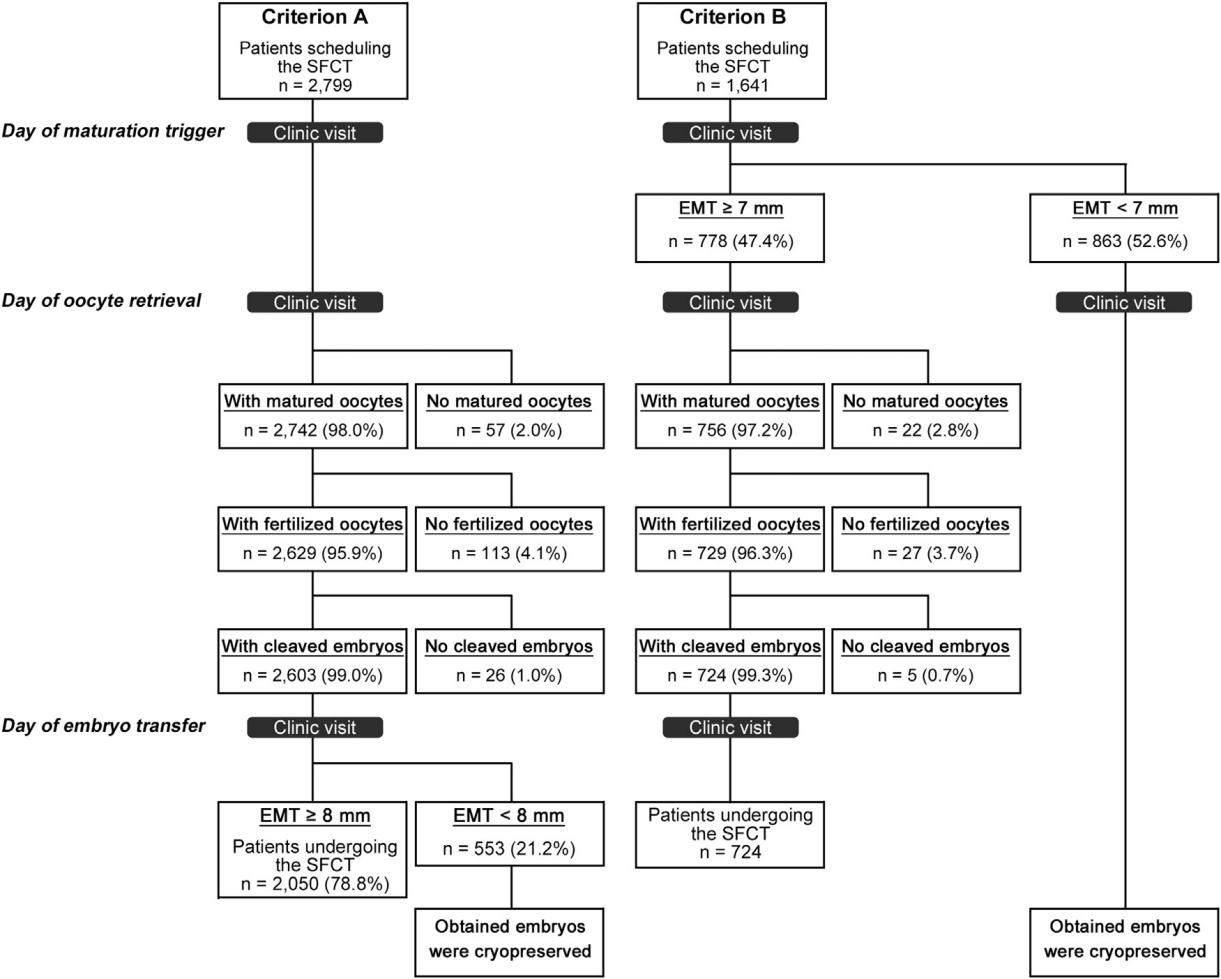
Flowchart describing the criteria for fresh embryo transfer The incidence of a thin endometrium was higher in the criterion B group (52.6%) than in the criterion A group (21.2%; *P*<.0001).

Nishii. Criteria based on endometrial thickness before trigger. Am J Obstet Gynecol Glob Rep 2023.

### Minimal ovarian stimulation cycle in in vitro fertilization

A detailed protocol for minimal stimulation with CC has been reported previously.^21^, ^22^, ^23^ Briefly, CC (50–100 mg/d) (Fuji Pharma Co, Ltd, Tokyo, Japan) was administered orally on an extended regimen from day 3 of the retrieval cycle to the day before induction of final oocyte maturation. EMT was measured using an ultrasound on the day of the trigger. When the dominant follicle developed to a size >18 mm, ovulation triggering was performed using a nasal spray containing the gonadotropin-releasing hormone agonist buserelin (Suprecur; Mochida Pharmaceutical Co, Ltd, Tokyo, Japan; or Buserecur, Fuji Pharma Co, Ltd).^24^

At 34 to 36 hours after triggering, oocyte retrieval was performed using a 21-gauge needle (Kitazato Corporation, Shizuoka, Japan), generally without anesthesia or follicular flushing.^25^ Cumulus-oocyte complexes (COCs) were collected, washed, and transferred to a human tubal fluid (HTF) medium (Kitazato Corporation) with paraffin oil at 5% CO_2_ in air at 37°C for culturing. Conventional IVF was performed 3 hours later. In cases of intracytoplasmic sperm injection (ICSI), denudation was performed at 4 hours after oocyte retrieval.^26^^,^^27^ For ICSI, cumulus cells surrounding the oocytes were removed, and the denuded oocytes were cultured in the HTF medium covered with paraffin oil for 1 hour before ICSI.

Sperm samples were collected by ejaculation and washed by centrifugation through 70% and 90% density gradients (Isolate; Irvine Scientific, Santa Ana, CA). The prepared sperm were cultured in HTF medium at 5% CO_2_ in air at 37°C until use.

### Conventional insemination, intracytoplasmic sperm injection, and embryo culture

For conventional IVF, HTF medium supplemented with 10% serum substitute (Irvine Scientific) was used as the fertilization medium.^26^ COCs were cultured with sperm (100,000 sperm/mL) at 5% CO_2_ in air at 37°C. Extrusion of the second polar body was confirmed at 5 hours after insemination (day 0) following the removal of cumulus cells. The oocytes were individually cultured in EmbryoSlide (Vitrolife, Göteborg, Sweden), which is suitable for group cultures in 180 µL of medium (ONESTEP medium; Nakamedical Inc, Tokyo, Japan) under paraffin oil. In cases of ICSI, oocytes were inseminated in HEPES-buffered HTF medium.^28^ Injected oocytes were immediately placed onto EmbryoSlides and cultured individually in 180 µL of medium in paraffin oil. Embryos were cultured at 37°C (gas phase, 5% O_2_, 5% CO_2_, and 90% N_2_) in an Embryoscope+ time-lapse incubator (Vitrolife) for 2 days. Fertilization was observed using the EmbryoViewer software (Vitrolife). Embryo vitrification and warming were performed using Cryotop (Kitazato Corporation) as described previously.^29^

### Embryo transfer

SFCT was performed on day 2 after oocyte retrieval as previously described.^20^^,^^24^ Dydrogesterone (30 mg per day orally) was routinely administered during the early luteal phase after embryo transfer. If luteal function was inadequate, progesterone was administered intravaginally (Lutinus; Ferring Pharmaceuticals, Saint-Prex, Switzerland) until week 9 of pregnancy. Clinical pregnancy was defined by observation of a gestational sac at 3 weeks after embryo transfer on an ultrasound scan, and ongoing pregnancy was defined by detection of a fetal heartbeat at 5 weeks after embryo transfer. The clinical pregnancy rate, ongoing pregnancy rate, and live birth follow-up data were analyzed.

### Statistical analyses

Statistical analyses were performed using the JMP software (SAS Inc, Cary, NC). Continuous parameters were compared using the Student *t* test or 1-way analysis of variance, and statistical significance was determined using the Tukey test for post hoc analysis. Proportion data were analyzed using the Pearson chi-square test. Logistic regression was used to assess the contributing strength of the parameters associated with pregnancy outcomes. Odds ratios (ORs) and adjusted ORs (AORs) are reported with 95% confidence intervals (CIs) for each group. Moreover, a receiver operating characteristic (ROC) analysis was performed, and the area under the ROC curve (AUC) was calculated. Statistical significance was set at *P*<.05.

## Results

### Patient characteristics

The characteristics of the criterion A and B groups are shown in Table 1 and the Figure. A total of 2799 patients in the criterion A group and 1641 patients in the criterion B group underwent oocyte retrieval. The SFCT cancellation rate was significantly higher in the criterion B group (55.9%) than in the criterion A group (26.8%; *P*<.0001), because the incidence of a thin endometrium was higher in the criterion B group (52.6%) than in the criterion A group (21.2%).

**Table 1:** Patient characteristics

Characteristics	Total	Criterion A^a^	Criterion B^a^	*P* value
Number of patients scheduled for SFCT, n	4440	2799	1641	
Number of patients who underwent fresh ET, n (%)	2774 (62.5)	2050 (73.2)	724 (44.1)	<.0001
Number of cancelled cycles, n (%)	1666 (33.8)	749 (26.8)	917 (55.9)	<.0001
Number of matured oocytes, n	79	57	22	
Number of fertilized oocytes, n	140	113	27	
Number of cleaved embryos, n	31	26	5	
Thin endometrium, n	1416	553	863	
Number of patients who underwent frozen ET, n	566	276	290	

*ET*, embryo transfer; *SFCT*, single fresh-cleaved embryo transfer.

a SFCT was performed when endometrial thickness on the day of SFCT was ≥8 mm (criterion A) or when endometrial thickness on the day of the trigger was ≥7 mm (criterion B).

### Correlation between endometrial thickness on the day of the trigger and live birth

The correlation of EMT on the day of the trigger with live birth in the criterion A group was examined (Table 2). The univariate and multivariate logistic regression analyses revealed a significant association between live birth rate and EMT on the day of the trigger (OR, 1.096; *P*=.0055; AOR, 1.098; *P*=.0110). However, no association was observed between the live birth rate and EMT on the day of SFCT.

**Table 2:** Multivariate logistic regression analysis of live birth rate

Characteristic	Univariate analysis				Multivariate analysis			
	OR	95% CI	*P* value	AUC	Adjusted OR	95% CI	*P* value	AUC
Female age	0.859	0.833–0.884	<.0001	0.678	0.866	0.836–0.897	<.0001	0.688
Male age	0.935	0.915–0.956	<.0001	0.601	0.989	0.965–1.014	.3841	
Number of previous embryo transfers	0.943	0.798–1.107	.4872	0.503	0.928	0.775–1.102	.4031	
Morphologic grade				0.522				
Grade 1	Reference	—	—		Reference	—	—	
Grade 2	0.859	0.626–1.178	.3472		0.949	0.681–1.321	.7552	
Grade 3	0.722	0.542–0.961	.0258		0.738	0.545–0.999	.0491	
Grade 4	0.000	—	.9853		0.000	—	.9973	
EMT on the day of the trigger	1.096	1.027–1.168	.0055	0.543	1.098	1.021–1.179	.0110	
EMT on the day of the transfer	1.053	0.998–1.110	.0547	0.542	0.993	0.934–1.053	.8243	

*AUC*, area under the curve; *CI*, confidence interval; *EMT*, endometrial thickness; *OR*, odds ratio.

### Modification of the criteria for single fresh-cleaved embryo transfer based on endometrial thickness

The outcomes of oocyte retrieval and embryo culture were comparable between the 2 criterion groups (Table S1). The demographic characteristics of the 2 criterion groups are shown in Table 3. Female and male age, number of previous embryo transfer cycles, and cause of infertility were comparable between the 2 groups. In addition, the serum levels of estradiol and progesterone on the day of the trigger were comparable between the 2 groups. However, EMT on the day of the trigger in the criterion B group was significantly greater than that in the criterion A group (*P*<.0001). In contrast, EMT on the day of SFCT was similar between the 2 groups. The insemination method and morphology of the transferred embryos were comparable between the 2 groups. Although the clinical pregnancy rate was comparable between the 2 criterion groups, the rates of ongoing pregnancy and live birth were significantly higher in the criterion B group than in the criterion A group (*P*=.0032 and *P*=.0281, respectively). The pregnancy outcomes were stratified by EMT on the day of the trigger and by the criteria for SFCT (Table 4). In both groups, the increased EMT was significantly correlated with live birth rate after SFCT in the CC-based minimal stimulation cycle (*P*=.0052 and *P*=.0422, respectively).

**Table 3:** Pregnancy outcomes after fresh embryo transfers on day 2

Characteristics	Criterion A	Criterion B	*P* value
Number of fresh embryo transfer cycles, n	2050	724	
Single embryo transfers, n (%)	970 (47.3)	323 (44.6)	.2099
Elective single embryo transfers, n (%)	1,080 (52.7)	401 (55.4)	
Female age, mean±SEM	37.9±0.1	37.7±0.1	.1154
Male age, mean±SEM	40.0±0.1	39.9±0.2	.7745
Number of previous oocyte retrieval cycles, mean±SEM	0.7±0.0	0.8±0.0	.1995
Number of previous embryo transfer cycles, mean±SEM	0.4±0.0	0.4±0.0	.0611
Number of patients with previous delivery, n (%)	441 (21.5)	173 (23.9)	.1843
Infertility cause, n (%)			.4127
Ovulation factor	49 (2.4)	12 (1.7)	
Oviduct factor	13 (0.6)	3 (0.4)	
Endometrial factor	236 (11.5)	72 (9.9)	
Male factor	618 (30.2)	254 (35.1)	
Combination	243 (11.9)	113 (15.6)	
Unexplained	891 (43.5)	270 (37.3)	
Serum estradiol on the day of the trigger (pg/mL)	878.4±7.7	900.8±13.8	.1452
Serum progesterone on the day of the trigger (ng/mL)	0.4±0.0	0.4±0.0	.3002
EMT on the day of the trigger (mm)	6.9±0.0	8.2±0.0	<.0001
EMT on the day of the transfer (mm)	10.4±0.0	10.5±0.1	.2836
Insemination			.4448
Conventional in vitro fertilization	746 (36.4)	275 (38.0)	
Intracytoplasmic sperm injection	1304 (63.6)	449 (62.0)	
Number of blastomeres of the transferred embryos, mean±SEM	5.7±0.0	5.8±0.1	.1996
Morphologic grade of the transferred embryos, n (%)			
Grade 1	379 (18.5)	136 (18.8)	
Grade 2	566 (27.6)	201 (27.8)	
Grade 3	1098 (53.6)	385 (53.2)	
Grade 4	7 (0.3)	2 (0.3)	
Clinical pregnancy, n (%)	509 (24.8)	203 (28.0)	.0892
Ongoing pregnancy, n (%)	411 (20.1)	185 (25.6)	.0019
Live birth, n (%)	393 (19.2)	167 (23.1)	.0248
Miscarriage, n (%)	116 (22.8)	36 (17.7)	.1372

*EMT,* endometrial thickness; *SEM,* standard error of mean.

**Table 4:** Pregnancy outcomes after fresh embryo transfers on day 2, stratified by endometrial thickness on the day of the trigger

Stratification criteria	Number of cycles	Female age	Clinical pregnancy (%)	Ongoing pregnancy (%)	Live birth (%)
**Criterion A**					
EMT: <6.0 mm	446	38.3±0.2	92 (20.6)^a^	75 (16.8)^a^	73 (16.4)^a^
EMT: 6.0–6.9 mm	514	37.8±0.2	119 (23.2)a,b	93 (18.1)a,b	87 (16.9)a,b
EMT: 7.0–7.9 mm	458	38.0±0.2	117 (25.6)a,b,c	91 (19.9)a,b,c	90 (19.7)a,b,c
EMT: 8.0–8.9 mm	365	37.6±0.2	101 (27.7)b,c	83 (22.7)b,c	82 (22.5)^c^
EMT: ≥9.0 mm	267	37.8±0.2	80 (30.0)^c^	69 (25.8)^c^	60 (22.5)b,c
*P* value	—	.1605	.0332	.0226	.0052
**Criterion B**					
EMT: 7.0–7.9 mm	303	38.0±0.2	73 (24.1)	68 (22.4)	61 (20.1)^a^
EMT: 8.0–8.9 mm	222	37.2±0.3	63 (28.4)	55 (23.9)	49 (22.1)a,b
EMT: ≥9.0 mm	199	37.7±0.3	67 (33.7)	62 (31.2)	57 (28.6)^b^
*P* value	—	.1189	.0647	.0865	.0422

*EMT*, endometrial thickness.

a–c Different superscript letters indicate significant difference at *P*<.05.

The pregnancy outcomes were stratified by both EMT on the day of the trigger (<7.0 mm vs ≥7.0 mm) and EMT on the day of SFCT (Tables S2 and S3). In the criterion A group, the live birth rate was significantly higher when EMT on the day of the trigger was ≥7.0 mm than when EMT on the day of the trigger was <7.0 mm (21.3% vs 16.7%) (Table S3). However, if EMT on the day of the trigger was ≥7.0 mm, the live birth rate was comparable between the criterion A and B groups (Table S2). Furthermore, although EMT on the day of SFCT was sufficient, the live birth rate tended to be lower when EMT on the day of the trigger was <7.0 mm than when EMT was ≥7.0 mm in all subgroups (Table S2). However, the rate of positive pregnancy outcomes were significantly lower when the EMT on the day of SFCT was <8 mm even if the EMT on the day of the trigger was ≥7.0 mm in the criterion B group (Table S3). Furthermore, in both the criterion A and B groups, the EMT increase between the days of the trigger and SFCT was not correlated with live birth rate (Table S4).

In cases in which SFCT was cancelled because of a thin endometrium, the embryos were vitrified and transferred in subsequent cycles. Table 5 shows the pregnancy outcomes after single vitrified-warmed cleaved embryo transfers. The insemination method, morphology of the transferred embryos, and patient characteristics were comparable between the 2 groups. The mean EMT on the day of the trigger was 8.4±0.1 mm in the criterion B group, which was comparable with that in the criterion A group. The rates of clinical pregnancy, ongoing pregnancy, and live birth were comparable between the groups.

**Table 5:** Pregnancy outcomes after frozen embryo transfers on day 2

Characteristics	Criterion A	Criterion B	*P* value
Number of frozen ET cycles, n	276	290	
Female age, mean±SEM (range)	39.2±0.2	38.6±0.2	.0949
Male age, mean±SEM (range)	41.2±0.3	40.5±0.3	.1364
Number of previous ET cycles, mean±SEM (range)	0.4±0.0	0.4±0.0	.4687
Endometrial thickness on the day of the trigger (mm)	8.3±0.1	8.4±0.1	.6091
Endometrial thickness on the day of the transfer (mm)	10.2±0.1	10.3±0.1	.2340
Insemination			.8502
Conventional in vitro fertilization	113 (40.9)	121 (41.7)	
Intracytoplasmic sperm injection	163 (59.1)	169 (58.3)	
Number of blastomeres of the transferred embryos, mean±SEM	5.8±0.8	5.8±0.8	.8228
Morphologic grade of the transferred embryos, n (%)			.3716
Grade 1	44 (15.9)	48 (16.6)	
Grade 2	79 (26.5)	94 (32.4)	
Grade 3	157 (56.9)	147 (50.7)	
Grade 4	2 (0.7)	1 (0.3)	
Clinical pregnancy, n (%)	75 (27.2)	91 (31.4)	.2720
Ongoing pregnancy, n (%)	63 (22.8)	79 (27.2)	.2258
Live birth, n (%)	56 (20.3)	68 (23.5)	.3639
Miscarriage*, n (%)*	19 (25.3)	24 (26.4)	.8790

*ET*, embryo transfer; *SEM*, standard error of mean.

The incidence of pregnancy complications tended to be lower in the criterion B group than in the criterion A group although there was no significant difference between the 2 groups (Table 6). However, a decreased incidence of placenta previa was observed in the criterion B group (Table 6). Furthermore, the multivariate logistic regression analysis demonstrated that an increased EMT on the day of the trigger was significantly correlated with a decreased incidence of placenta previa (Table 7).

**Table 6:** Maternal complications during the perinatal period

Maternal complications	Criterion A	Criterion B	*P* value
Deliveries, n	393	167	
Pregnancy complications, n (%)	38 (9.7)	8 (4.8)	.0544
Hypertensive disorders of pregnancy, n (%)	7 (1.8)	3 (1.8)	.9901
Gestational diabetes mellitus, n (%)	4 (1.0)	2 (1.2)	.8500
HELLP syndrome, n (%)	4 (1.0)	1 (0.6)	.6297
Preterm premature rupture of membranes, n (%)	3 (0.8)	0 (0)	.2576
Low-lying placenta, n (%)	1 (0.3)	1 (0.6)	.5320
Placenta previa, n (%)	17 (4.3)	1 (0.6)	.0222
Placental abruption, n (%)	3 (0.8)	0 (0)	.2576
Other, n (%)	1 (0.3)	0 (0)	.5141

*HELLP*, hemolysis, elevated liver enzymes and low platelet count.

**Table 7:** Multivariate logistic regression analysis for the incidence of placenta previa

Characteristics	Univariate analysis				Multivariate analysis			
	Odds ratio	95% CI	*P* value	AUC	Adjusted odds ratio	95% CI	*P* value	AUC
Female age	1.01	0.88–1.15	.8623	0.484	—	—	—	0.709
Male age	1.01	0.92–1.11	.7372	0.529	—	—	—	
Body mass index	1.27	1.06–1.52	.0074	0.654	1.28	1.06–1.55	.0094	
Number of previous delivery	1.31	0.53–3.22	.5441	0.518	—	—	—	
Morphologic grade								
Grade 1	Reference	—	—	0.535	—	—	—	
Grade 2	1.49	0.36–6.12	.5721		—	—	—	
Grade 3	1.30	0.34–4.90	.6938		—	—	—	
EMT on the day of the trigger	0.67	0.49–0.90	.0101	0.668	0.67	0.45–0.98	.0387	

*AUC*, area under the curve; *CI*, confidence interval; *EMT*, endometrial thickness.

## Discussion

### Principal findings

This study revealed that EMT on the day of the trigger was significantly associated with live birth rate after SFCT in a CC-based minimal stimulation cycle. Furthermore, the use of EMT on the day of ovulation trigger as a criterion for SFCT significantly improved the live birth rate, contrary to the use of EMT on the day of embryo transfer.

### Result and clinical implications

Our recent study reported that EMT on the day of the trigger was correlated with the ongoing pregnancy rate after SFCT in a CC-based minimal stimulation cycle, suggesting that EMT on the day of the trigger may be predictive of pregnancy outcomes. However, live birth rate after SFCT was not examined in the previous study.^20^ In this study, we further showed that EMT on the day of the trigger was also associated with live birth rate after SFCT but not with EMT on the day of SFCT. Furthermore, our results showed that the live birth rate tended to be lower when EMT on the day of the trigger was thin (<7.0 mm) despite EMT being sufficient on the day of the transfer. The functional layer is thickened under the influence of estrogen in proliferation phases, and progesterone inhibits estrogen-induced proliferation and promotes decidualization in the secretory phase.^1^^,^^30^ Therefore, the increase in EMT on the day of the trigger is affected by estrogen, reflecting the degree of proliferation (quantity of endometrium). The increase in EMT on the day of SFCT is affected by both estrogen and progesterone, reflecting the degree of differentiation (endometrium quality)^1^ and proposing that the biological significance of increased EMT is different between the proliferation and secretory phases. Therefore, our results suggest that the estrogen-induced EMT increase during the proliferation phase is more crucial for the success of implantation and live birth than the EMT increase during the secretory phase. Nevertheless, further studies are required to explain why EMT on the day of trigger provides a better prediction of live birth.

From our results, it was hypothesized that modification of the indication for SFCT may lead to an improvement in the live birth rate after SFCT. By modifying the criteria for SFCT based on EMT on the day of the trigger (criterion B, EMT ≥7 mm), the rates of ongoing pregnancy and live birth rate significantly improved when compared with the rates obtained when the previous criterion was used (criterion A, EMT ≥8 mm on the day of embryo transfer). These results clearly demonstrated that modifying the SFCT criteria could lead to an improvement in pregnancy outcomes. Furthermore, when EMT was <7 mm on the day of the trigger, oocyte retrieval was performed, and the obtained embryos were vitrified and used for frozen embryo transfers in subsequent cycles. In these patients, EMT was sufficiently thickened under the CC-free environment in the next cycle, and the live birth rate after frozen embryo transfers (23.5%) (Table 5) was comparable with that of patients who exhibited an EMT of ≥7.0 mm on the day of the trigger in the criterion A and B groups (21.3% and 22.9%, respectively) (Table S2). These results suggest that the cause of poor pregnancy outcomes observed when EMT was <7 mm on the day of the trigger was not because of low embryo competence, but rather the EMT. Furthermore, if these embryos were transferred in the oocyte retrieval cycle, the expected live birth rate was 16% to 17%; therefore, if EMT is <7 mm on the day of the trigger, fresh embryo transfers should be cancelled, and frozen embryo transfers should be rescheduled to improve the live birth rate per obtained embryo.

Furthermore, by investigating the maternal outcomes, we found that the incidence of placenta previa in the criterion B group was significantly lower than that in the criterion A group. The pathophysiology of placenta previa is not well understood; however, altered endometrial blood flow and abnormal uterine contractions have been proposed as common mechanisms.^31^ Our data also showed that placenta previa frequently occurred when the EMT on the day of the trigger was thin. Considering that the EMT on the day of the trigger in the criterion B group was significantly greater than that in the criterion A group, modifying the criteria for SFCT based on the EMT on the day of the trigger might reduce the risk of placenta previa by cancelling the embryo transfer cycles when the endometrial blood flow and subsequent proliferation were insufficient.

### Strengths and limitations

The main strength of this study was that the data were obtained from a single-center cohort of patients who underwent SFCT in a CC-based minimal stimulation cycle; therefore, the treatment strategy and protocol used were uniform. However, several limitations should be mentioned. The study was retrospective in nature and was performed in a single center; therefore, further multicenter studies are required to ascertain the generalizability of these findings to other clinics with different patient demographics and/or protocols, such as the COS cycle.

## Conclusion

This study showed a significant association between a decreased EMT on the day of the trigger and a low live birth rate. In addition, EMT on the day of trigger is associated with a risk for placenta previa. We observed that modifying the SFCT criteria to reflect EMT on the day of the trigger would improve pregnancy outcomes; this implies that the criteria for SFCT are no longer based on the day of transfer, but rather on the day of the trigger. Furthermore, this modification may reduce patient burden by reducing the number of unnecessary clinic visits and the cost of embryo transfer under poor conditions. Therefore, EMT on the day of the trigger should be included in the criteria for embryo transfer to improve clinical outcomes.

## Appendix Supplementary materials

Image, application 1
