# Supplementary material for: Association between endometrial thickness before ovulation, live birth, and placenta previa rates in clomiphene citrate-treated cycles
Source: AJOG Glob Rep. 2023 Jan 18;3(1):100161. doi: 10.1016/j.xagr.2023.100161 (PMC9975686; doi:10.1016/j.xagr.2023.100161)
Supplement: Supplementary file 1 [file mmc1.docx]

**Table S1. Outcomes of in vitro fertilization in the cycles with fresh embryo transfers**

|  | **Criterion A** | **Criterion B** | ***P* value** |
| --- | --- | --- | --- |
| No. of cycles, *n* | 2,050 | 724 |  |
| No. of fully developed antral follicles at the trigger, mean ± SEM | 2.9 ± 0.0 | 2.9 ± 0.0 | 0.2022 |
| No. of retrieved oocytes, mean ± SEM | 2.2 ± 0.0 | 2.2 ± 0.0 | 0.1471 |
| No. of matured oocytes, mean ± SEM | 2.0 ± 0.0 | 2.1 ± 0.0 | 0.1294 |
| No. of fertilized oocytes, mean ± SEM | 1.8 ± 0.0 | 1.8 ± 0.0 | 0.1146 |
| No. of cleaved embryos, mean ± SEM | 1.7 ± 0.0 | 1.8 ± 0.0 | 0.1010 |
| Inseminated oocytes, *n* | 4,173 | 1,520 |  |
| Fertilized oocytes, *n* (%) | 3,641 (87.3) | 1,331 (87.6) | 0.7523 |
| Cleaved embryos, *n* (%) | 3,582 (85.8) | 1,312 (86.3) | 0.6458 |
| No. of blastomeres on day 2, mean ± SEM | 4.1 ± 0.0 | 4.1 ± 0.0 | 0.4007 |
| Morphological grade of the cleaved embryos, *n* (%) |  |  | 0.0819 |
| Grade 1 | 458 (12.8) | 163 (12.4) |  |
| Grade 2 | 1,089 (30.4) | 415 (31.6) |  |
| Grade 3 | 1,943 (54.2) | 716 (54.6) |  |
| Grade 4 | 92 (2.6) | 18 (1.4) |  |
| No. of patients with ≥2 embryos | 1,080 (52.7) | 401 (55.4) | 0.2099 |

*SEM,* standard error of mean

**Table S2. Pregnancy outcomes after fresh embryo transfers on day 2, stratified by the endometrial thickness on the day of the trigger**

| **EMT on the trigger day** | **EMT on the transfer day** | **No. of cycles** | **Female age** | **Clinical pregnancy (%)** | **Ongoing pregnancy (%)** | **Live birth (%)** |
| --- | --- | --- | --- | --- | --- | --- |
| **Criterion A** |  |  |  |  |  |  |
| EMT: <7.0 mm | EMT: 8.0–8.9 mm | 210 | 39.2 ± 0.3^a^ | 29 (13.8)^a^ | 23 (11.0)^a^ | 22 (10.5)^a^ |
|  | EMT: 9.0–9.9 mm | 234 | 38.0 ± 0.3^b^ | 56 (23.9)^b^ | 44 (18.8)^b^ | 42 (18.0)^b^ |
|  | EMT: 10.0–10.9 mm | 333 | 37.9 ± 0.2^b^ | 79 (23.7)^b^ | 64 (19.2)^b^ | 61 (18.3)^b^ |
|  | EMT: ≥11.0 mm | 183 | 37.0 ± 0.3^b^ | 47 (26.7)^b^ | 37 (20.2)^b^ | 35 (19.1)^b^ |
|  | Total | 960 | 38.0 ± 0.1 | 211 (22.0)^a^ | 168 (17.5)^a^ | 160 (16.7)^a^ |
| EMT: ≥7.0 mm | EMT: 8.0–8.9 mm | 104 | 38.5 ± 0.4^a^ | 25 (24.0) | 19 (18.3) | 19 (18.3) |
|  | EMT: 9.0–9.9 mm | 111 | 38.4 ± 0.4^a^ | 27 (24.3) | 23 (20.7) | 23 (20.7) |
|  | EMT: 10.0–10.9 mm | 391 | 38.1 ± 0.2^a^ | 102 (26.1) | 83 (21.2) | 81 (20.7) |
|  | EMT: ≥11.0 mm | 484 | 37.3 ± 0.2^b^ | 144 (29.8) | 118 (24.4) | 109 (22.5) |
|  | Total | 1,090 | 37.8 ± 0.1 | 298 (27.3)^b^ | 243 (22.3)^b^ | 232 (21.3)^b^ |
| **Criterion B** |  |  |  |  |  |  |
| EMT: ≥7.0 mm | EMT: <9.0 mm | 130 | 38.8 ± 0.4^a^ | 25 (19.2) | 23 (17.7) | 22 (16.9) |
|  | EMT: 9.0–9.9 mm | 74 | 38.4 ± 0.5^a^ | 19 (25.7) | 18 (24.3) | 15 (20.3) |
|  | EMT: 10.0–10.9 mm | 266 | 37.8 ± 0.2^a^ | 77 (29.0) | 68 (25.6) | 65 (24.4) |
|  | EMT: ≥11.0 mm | 254 | 36.7 ± 0.2^b^ | 80 (31.5) | 74 (29.1) | 64 (25.2) |
|  | Total | 724 | 37.7 ± 0.1 | 201 (27.8)^b^ | 183 (25.3^)b^ | 166 (22.9)^b^ |

*EMT,* endometrial thickness

^a–b^ Different superscript letters indicate significant difference at *P* < 0.05

**Table S3. Pregnancy outcomes after fresh embryo transfers on day 2, stratified by the endometrial thickness on the day of the trigger and embryo transfer**

| **EMT on the trigger day** | **EMT on the transfer day** | **No. of cycles (%)** | **Female age** | **Clinical pregnancy (%)** | **Ongoing pregnancy (%)** | **Live birth (%)** |
| --- | --- | --- | --- | --- | --- | --- |
| **Criterion A** |  |  |  |  |  |  |
| EMT: <7.0 mm | EMT: ≥8.0 mm | 960 (46.8) | 38.0 ± 0.1 | 211 (22.0)^a^ | 168 (17.5)^a^ | 160 (16.7)^a^ |
| EMT: ≥7.0 mm | EMT: ≥8.0 mm | 1,090 (53.2) | 37.8 ± 0.1 | 298 (27.3)^b^ | 243 (22.3)^b^ | 232 (21.3)^b^ |
| **Criterion B** |  |  |  |  |  |  |
| EMT: ≥7.0 mm | EMT: <8.0 mm | 18 (2.5) | 39.4 ± 0.9^a^ | 1 (5.6)^a^ | 1 (5.6)^a^ | 0 (0)^a^ |
| EMT: ≥7.0 mm | EMT: ≥8.0 mm | 706 (97.5) | 37.6 ± 0.1^a^ | 202 (28.6)^b^ | 184 (26.1)^b^ | 167 (23.7)^b^ |

*EMT,* endometrial thickness

^a–b^ Different superscript letters indicate significant difference at *P* < 0.05

**Table S3. Pregnancy outcomes after fresh embryo transfers on day 2, stratified by the increase in endometrial thickness between the days of the trigger and embryo transfer**

| **EMT increase between the days of the trigger and embryo transfer** | **Criterion A** |  |  | **Criterion B** |  |
| --- | --- | --- | --- | --- | --- |
|  | **No. of cycles** | **Live birth (%)** |  | **No. of cycles** | **Live birth (%)** |
| EMT increase: <1.0 mm | 135 | 27 (20.0) |  | 117 | 25 (21.4) |
| EMT increase: 1.0–1.9 mm | 188 | 40 (21.3) |  | 160 | 34 (21.3) |
| EMT increase: 2.0–2.9 mm | 387 | 71 (18.4) |  | 170 | 41 (24.1) |
| EMT increase: 3.0–3.9 mm | 462 | 86 (18.6) |  | 152 | 34 (22.4) |
| EMT increase: 4.0–4.9 mm | 380 | 71 (18.7) |  | 46 | 13 (28.3) |
| EMT increase: ≥5.0 mm | 498 | 97 (19.5) |  | 79 | 19 (24.1) |
| *P* value |  | 0.9667 |  |  | 0.9269 |

*EMT,* endometrial thickness
